# Current Approaches to Diagnosis of Early Proximal Carious Lesion: A Literature Review

**DOI:** 10.7759/cureus.43489

**Published:** 2023-08-14

**Authors:** Abdulrahman D Al Saffan

**Affiliations:** 1 Preventive Dentistry Department, College of Medicine and Dentistry, Riyadh Elm University, Riyadh, SAU

**Keywords:** review article, methods, detection, diagnosis, proximal caries, incipient carious lesions

## Abstract

Integrating technological tools with clinical visual examination for caries detection and diagnosis can improve preventative measures in dentistry, resulting in decreased treatment expenses and reduced time and costs associated with testing potential anticaries agents. This article provides an overview of the conventional and new emerging modern technologies that can assist dental professionals in the early detection and diagnosis of dental caries. These technologies aid in assessing the progression of carious lesions and monitoring them quantitatively or qualitatively over time. Traditional techniques (visual, tactile, and radiographic) have limitations in diagnosing early proximal caries accurately. Novel methods like fluorescence and transillumination, as well as advanced tools like OCT (optical coherence tomography), laser fluorescence, and QLF (quantitative light-induced fluorescence), are effective for early caries detection. Optical methods like fluorescence and transillumination are particularly successful in identifying initial caries stages. Moreover, this review highlights the clinical relevance of these methods and discusses potential future technologies like terahertz imaging and artificial intelligence (AI)-based approaches.

## Introduction and background

Dental caries and periodontal disease have traditionally been regarded as the primary oral health challenges on a global scale. Dental caries remains a significant health concern in most developed nations, affecting 60-90% of school-aged children and the overwhelming majority of adults. The prevalence of dental caries in children is comparatively higher in the American (DMFT [decayed, missing, and filled permanent teeth] index = 3.0) and the European regions (DMFT = 2.6), while it is lower in the majority of African countries (DMFT = 1.7). Globally, there is a significant prevalence of dental caries among adults, affecting nearly 100% of the population in most countries. Nevertheless, a substantial proportion of adult populations worldwide, ranging from 5% to 20%, exhibit the presence of severe periodontitis, a condition that can ultimately lead to tooth loss. The considerable impact of pain and suffering, impairment of function, and reduced quality of life caused by the high prevalence of dental caries were observed in both individuals and communities [1].

Dental caries is a highly prevalent chronic disease of the teeth that affects individuals globally. When considering the inclusion of initial lesions in the clinical assessment, it is evident that only a few individuals remain unaffected by dental caries [1]. In recent years, there has been a noticeable shift in the patterns of dental caries progression. The rate of advancement of non-cavitated lesions appears sluggish [2]. This characteristic enables the implementation of preventive measures when the lesions are most likely to be halted. Integrating conventional techniques with more advanced and precise methods can enhance the diagnosis of caries and facilitate the monitoring of non-surgical treatments by clinicians [3].

Failure to detect lesions in the early stages could be the reason for not implementing the effective caries preventive measures [4]. Previous workshops and conferences on dental caries have emphasized the challenge of accurately detecting early caries lesions and the necessity of implementing a revised system based on caries risk, lesion severity, surface integrity, and caries activity for the non-surgical treatment of non-cavitated lesions [5]. Caries lesion detection entails an objective technique for determining the disease's presence or absence [6]. The objective of lesion assessment is to characterize or monitor a detected lesion [3], and the diagnosis of caries should encompass a summation of all pertinent data by a qualified healthcare professional [7]. 

Interproximal caries lesions are formed between the proximal surfaces of two neighboring teeth. Initially, these surfaces manifest as opaque areas clinically due to the diminished translucency of enamel in the outermost layer between the contact point and the uppermost part of the free gingival margin [8,9]. Due to the considerable dimensions of the proximal areas of posterior teeth as well as inconspicuous demineralization that characterizes early-stage lesions on these surfaces, detecting proximal caries on posterior teeth is typically challenging when relying solely on radiographic examination [10,11].

When assessing the diagnostic value of dental caries tests, it is crucial to consider the sensitivity and specificity of the trial, along with the predictive values. Sensitivity refers to the accuracy of a test in correctly identifying true positives, while specificity refers to the accuracy of difficulty in correctly identifying true negatives. A positive predictive value refers to the proportion of individuals who receive positive test results and are correctly diagnosed, while a negative predictive value refers to the ratio of individuals who receive negative test results and are accurately diagnosed [12,13]. When it comes to detecting and diagnosing caries, tests that exhibit high sensitivity can accurately identify the presence of caries. Similarly, tests that exhibit high specificity will accurately detect the absence of caries.

In contemporary times, there is a growing recognition of the significance associated with the early detection and diagnosis of carious lesions to prevent their progression into irreversible cavitation. Many dental professionals encounter challenges when determining the appropriate timing for implementing preventive measures versus intervening in dental procedures. Assessment of the patient's caries activity is essential in diagnosing and formulating an appropriate treatment plan. Individuals with low caries activity may exhibit more efficient repair mechanisms, thus potentially allowing for the healing of white spot lesions. Designing the treatment protocol based on the caries activity after an accurate diagnosis is crucial. Therefore, the primary objective of this review is to report the conventional and contemporary approaches utilized in detecting and diagnosing initial proximal caries lesions.

## Review

Conventional methods

The conventional techniques used for assessing caries through visual examination and tactile perception and their interpretation with radiographs are commonly referred to as traditional methods [14].

Visual Examination

Visual examination is the diagnostic method most commonly employed in the daily practice of dentistry [15]. Determining lesion activity, whether active or inactive, can be achieved through a visual examination of white spot lesions. Chalky and rough surfaces suggest an active lesion, whereas smooth and shiny surfaces indicate an inactive lesion [16]. One notable benefit of this approach is its simplicity, cost-effectiveness, and solid clinical validity. On the other hand, a significant disadvantage of this approach is the difficulty in achieving standardization [17].

Visual and Tactile Method

The identification of dental caries has traditionally relied heavily on visual examination and clinical inspection. Additional tactile data can be acquired using a dental mirror, light source, explorer, or probe after teeth cleaning and drying procedures. These aspects depend on the dentists' subjective interpretation of visual clues [18,19]. However, visual-tactile inspection restricts the examiner's ability to evaluate solely the surfaces of teeth readily accessible in a clinical situation [20]. Previous studies on identifying carious lesions through visual and tactile inspections have yielded inconsistent results. These discrepancies can be attributed to the utilization of diverse criteria for visual inspection and the variability in the conditions in which the examinations are conducted [21].

International Caries Detection and Assessment System (ICDAS I)

There was a push to develop validated systems for caries detection to address the inconsistent criteria used for caries diagnosis. The ICDAS I was developed in 2001 that utilizes a visual scoring method to detect caries [21]. Recent studies have demonstrated that implementing meticulous and validated techniques for the visual examination of dental caries has yielded good accuracy. Specifically, these studies have indicated a higher specificity level than sensitivity in detecting carious lesions [22]. Multiple classifications exist for white spot lesions, with the ICDAS classification being the most commonly employed. This classification system was developed by Ekstrand et al. and incorporates the most effective attributes from various detection and evaluation systems utilized for caries detection [23]. However, several barriers were identified in implementing the ICDAS criteria in routine dental practice. These barriers include time constraints, inadequate training, limited impact on treatment planning, challenges in charting, and low patient awareness regarding preventive measures [24].

Radiographic method

While visual examination demonstrates a high specificity for detecting caries on visible surfaces of teeth, the detection of approximal carious lesions is only around 0.30, meaning that about 70% of cavitated caries lesions would be undetected [25]. Radiographic caries detection, used alone or in conjunction with clinical assessment, is the most common diagnostic tool by the dentists along with visual-tactile screening [26]. Adding bitewing radiography as an adjuvant to visual examination allows for more sensitive detection of proximal and occlusal caries lesions, providing a better estimation of lesion depth than visual inspection alone. Moreover, it permits monitoring of the lesion's progress over time [27].

International Caries Detection and Assessment System (ICDAS II)

In 2004, the ICDAS I was revised to incorporate radiographic images of enamel and dentin lesions which is known as ICDAS II. This modification involved the classification of lesions into five distinct classes: E1: Lesion that reaches the outer half of the enamel; E2: Lesion that has advanced to the inner half of the enamel; D1: Lesion that is limited to the outer one-third of the dentin; D2: Lesion that has advanced to the middle third of the dentin; and D3: Lesion that has advanced to the inner one-third of the dentin [28]. It has been demonstrated that radiographic caries detection exhibits lower sensitivity but higher specificity when identifying initial proximal caries lesions [29]. Consequently, there is a notable occurrence of false-negative outcomes, which increases the risk of disease progression at an early stage [30]. 

According to the WHO criteria, the presence of caries is documented when a lesion is observed in the pit and fissure or on a smooth tooth surface, displaying a clear cavity, undermined enamel, or a detectably softened floor or wall. In cases of uncertainty, it is advisable not to document the presence of caries. The research findings indicated a significant variation in the occurrence of dental caries when comparing the ICDAS II, and WHO approaches to diagnosing caries. The identification of non-cavitated carious lesions was alarming. Using the ICDAS II criteria for caries diagnosis, as opposed to the WHO criteria, has demonstrated its value in the timely identification and intervention of early/non-cavitated carious lesions [31]. Moreover, DIAGNOdent performance was comparable to that of ICDAS II visual inspection in terms of early caries detection [32]. 

In recent years, several novel technologies have emerged, leading to the development of various new tools that facilitate the early detection of caries. These devices are designed to assist healthcare professionals in establishing a definitive diagnosis and enabling prompt and conservative therapeutic interventions. The devices employ various technologies such as fluorescence, reflectance, and electrical conductance or impedance to assess the demineralization of enamel and enable the tracking of lesion progression over time.

Contemporary methods

Transillumination

Transillumination is among the earliest techniques to diagnose dental caries [33]. This instrument proves advantageous, particularly in identifying and assessing approximal caries [4]. The transillumination method depends on the fundamental principle that a difference in light transmittance exists between carious and healthy tooth tissues. The structure of solid enamel is characterized by the interlocking arrangement of hydroxyapatite crystals, resulting in a translucent appearance [34]. Demineralization leads to the degradation of the enamel structure. In instances of this nature, the incident light's photons undergo scattering, resulting in a decline in the optical characteristics of the enamel. The presence of a decayed tooth structure results in light entrapment, leading to a reduction in light permeability compared to intact enamel.

Consequently, when exposed to light, various regions within the tooth manifest as dark shadows during a clinical examination [35]. The transillumination method has proven to be a promising technique in dentistry in caries identification. Various technologies have been developed utilizing this principle [4,33,36]. These include the fiber optic transillumination (FOTI) method and the digital fiber optic transillumination (DIFOTI) system that emerged, combining the utilization of the FOTI technique with digital recording systems. Following the advancement of fiber optics utilizing visible light for early caries detection, the near-infrared light transillumination (NILT) technique has been devised to facilitate the transillumination of teeth. This method combines near-infrared (NIR) light and digital camera systems, offering enhanced light penetration capabilities [35,36].

FOTI: Caries detection through the use of FOTI involves the utilization of a light source of high intensity, which can be conveniently and flexibly employed in any region of the oral cavity. The underlying mechanism of this technique is based on the premise that carious dental tissue exhibits a distinct light transmission index compared to healthy dental tissue. The presence of demineralized regions in enamel and dentin is correlated with a reduced level of light transmission, resulting in a darkened shadow corresponding to the progression of dental decay [37]. The light probe is positioned apically beyond the cervical margin along the gingiva to detect proximal caries in the premolar and molar teeth. The transmission of light through dental structures allows for the visualization of decay from the occlusal perspective as a shadow. In most instances, the shadow accurately depicts the magnitude of the compromised dental structure affected by caries [37].

Furthermore, it is possible to position the light source at various angles relative to the tooth, enabling a three-dimensional assessment of caries penetration [38]. Recent advancements in FOTI have introduced a novel component consisting of a slender and pliable fiber-optic tip. This innovative tip is designed to be inserted into the gingival embrasure, specifically beneath the proximal contact and adjacent to the marginal ridge. Using a 0.75 mm diameter tip enhances the visibility of caries compared to conventional fiber optic tips [39]. Using FOTI in conjunction with visual and radiographic assessment techniques can enhance the identification of carious lesions [40]. The intensity shadows created by the device's light output may be used to differentiate early caries lesions [17].

DIFOTI: DIFOTI was developed to overcome the irregularities in the visual inspection of FOTI within and between observers. DIFOTI-enabled CCD (charge-coupled device) cameras collect and record pictures instantly. Digitally processed pictures of anomalies or lesions may be seen on a monitor [41]. There was a belief that DIFOTI could enhance the early identification of various surfaces. In vitro, methods found that DIFOTI exhibited a high level of sensitivity in detecting lesions during the initial stages of demineralization. However, it was unable to accurately measure the depth of the lesion when compared to histologic analysis and surface cavitation [42]. However, a stronger correlation was observed for smaller lesions [43]. Another study demonstrated that DIFOTI outperformed radiography in detecting early or small lesions, while its performance was comparable to radiography for more extensive lesions that penetrate the dentin [44].

NILT: Building upon the advancements in FOTI (fluorescence optical imaging) and DIFOTI techniques that utilize visible light, a novel technology has surfaced that employs NIR light to transilluminate the tooth. The DIAGNOcam, developed by KaVo in Biberach, Germany, was introduced in 2012. This camera system utilizes an NIR light source. Light transmission occurs through the gingiva, alveolar bone, and root structures, ultimately reaching the crown - a carious lesion results in the dispersion and attenuation of transmitted light. A CCD sensor captures clinical data and generates a tooth image as observed from its occlusal surface [45]. 

Photo-optical caries detection was initially described in 1995 and was improvised later based on technological innovations. The wavelengths of light within the NIR spectrum, which spans from 700 to 1500 nm, exhibit significantly greater length than those found within the visible light range. Light with longer wavelengths demonstrates reduced scattering and a remarkable ability to penetrate objects at greater depths [46]. Research findings have provided evidence that the application of NIR transillumination to teeth results in the manifestation of enamel transparency. This technique enables the illumination of the tooth's buccal surface and the simultaneous visualization of occlusal as well as proximal caries lesions [47]. The NILT imaging system enables the visualization of the complete occlusal surface [48].

In addition, the absence of ionizing radiation in this method eliminates any limitations on the acquisition of images. The utilization of NILT has demonstrated its potential as a valuable screening tool in the timely identification of hidden demineralized lesions. Moreover, it can be regarded as a supplementary method to bitewing radiographs. The utilization of this approach may offer benefits in the screening of pregnant individuals, as well as in the assessment of growing adolescents. Additionally, it is valuable in situations requiring frequent monitoring [49,50]. Additionally, NILT has demonstrated the potential efficacy in the detection of early caries in the proximal surfaces. Moreover, proximal caries detection accuracy was similar between NILT technology and digital radiography [51].

Optical Coherence Tomography (OCT)

Using infrared light in the wavelength of 1310 nm, OCT creates cross-sections of biological tissues without producing ionizing radiation [52,53]. OCT is an optical imaging technique that can conduct non-invasive tomographic imaging with micrometers resolution. Following its initial introduction in 1991 in the United States, the acceptance and endorsement of dentists and health authorities worldwide took over a decade to materialize it. 

The utilization of OCT in identifying minute alterations in the visual and morphological characteristics of tooth surfaces while avoiding the need for ionizing radiation confers an additional safety benefit for both patients and clinicians. The integration of OCT with diverse image processing methodologies has been the subject of extensive investigation. This integration can potentially provide dental professionals with enhanced objectivity in diagnosing demineralized lesions of teeth [54].

The OCT is a sophisticated and non-invasive imaging technique that utilizes NIR light to visualize and evaluate tissue samples through interferometric means. In dentistry, OCT has demonstrated its capacity to effectively visualize the initial stages of tooth demineralization and the subsequent development of caries in living organisms without requiring ionizing radiation. In addition, OCT can exhibit comparable morphological and optical alterations in both intact and demineralized teeth, akin to other sophisticated technologies like micro-computed tomography (µCT) [54,55].

Fluorescent Techniques

Laser fluorescence: Laser fluorescence refers to the emission of light by a material after it has absorbed photons from a laser source. The DIAGNOdent, which was introduced by KaVo in 1998, employs laser fluorescence as a means of identifying caries. The device utilizes a diode laser that emits a beam of 655 nanometers wavelength. When applied to a tooth's surface, the wavelength in question is absorbed by the metabolic byproducts of bacteria and subsequently emits red fluorescence. The degree of fluorescence reflected is quantified by a numerical value in the range of 0-99 on the device's display. A higher score corresponds to a more significant decay extent [56].

A positive linear relationship exists between the carious lesion extent and the corresponding DIAGNOdent reading. The value of the lesion will experience a gradual increase as long as it remains limited to the enamel. However, there will be a significant increase in value once the lesion surpasses the dentinoenamel junction. Additionally, it has been demonstrated that DIAGNOdent exhibits considerable consistency among examiners, indicating its potential as a viable approach for the ongoing assessment of dental caries over time. Similarly, utilizing this approach can prove advantageous in assessing the results of preventive interventions [57].

Moreover, the laser fluorescence method demonstrates the highest sensitivity and diagnostic accuracy among all available methods for early detection of proximal caries. The higher prevalence of false-positive results obtained from DIAGNOdent suggests that it is more appropriate to utilize the device as a supplemental tool to enhance the diagnostic precision of traditional detection methods rather than relying solely on it as the only method for early caries detection. The DIAGNOdent examination can be repeated multiple times, as it is considered safe, non-invasive, and highly reproducible [58]. Recent research findings indicated that the ICDAS II and DIAGNOdent methods could identify the remineralization of initial enamel occlusal caries. The DIAGNOdent method exhibited greater sensitivity in detecting remineralization compared to the histological scores and the ICDAS II method [59].

Quantitative light-induced fluorescence (QLF): The development of this novel technology in 2003 was founded on the optical principles of fluorescence, enabling the calculation of a quantitative measurement for the disparity in fluorescence radiance between tooth structures affected by caries and healthy ones [60]. QLF is a method that utilizes the inherent fluorescence of teeth, which is known to diminish in the presence of demineralization [61]. When observed using QLF, carious lesions will exhibit a dark appearance. This can be attributed to the principle that demineralized tissue restricts the passage of light by causing an excessive scattering of photons [62].

The QLF enables the easy detection of lesions measuring 500 micrometers in depth on both smooth and occlusal surfaces. The technology has shown significant potential in terms of its ability to detect early caries with high sensitivity and reproducibility. Additionally, it has demonstrated effectiveness in monitoring the progression or regression of lesions over time [63]. An enhanced iteration of the device, commonly referred to as Biluminatorᵀᴹ (QLF-D), has been developed. This device demonstrated high effectiveness and comparability to conventional techniques in identifying proximal caries [64]. The QLF-D system can quantitatively identify mineral loss and detect red fluorescence indicative of the biofilm, facilitating the identification of an individual's caries risk status and the subsequent development of suitable preventive measures [65].

Recent studies have shown that the comparative reliability of QLF in the detection of primary teeth caries was found to be on par with, or marginally superior to, conventional diagnostic techniques such as visual inspection or radiographic examination under clinical circumstances [66-68]. Using QLF technology has proven to be a reliable approach for diagnosing early carious lesions and monitoring the effectiveness of treatment interventions. However, it should be coupled with other diagnostic methods [19]. 

Light-Emitting Diode (LED)

The Midwest Caries I.D., developed by DENTSPLY Professional in York, PA, is a portable device that employs the principles of LED reflectance and refraction technology [69]. This device can identify variations in optical properties within the tooth associated with alterations in its structural composition. It employs infrared- and red-light-emitting diodes and an optical fiber to deliver light to the specific region examined at the probe's tip. A secondary optical fiber is used to capture light emanating from the observed area, which is then directed toward a photodetector responsible for quantifying the intensity of the collected light. Subsequently, the photodetector signal is transmitted to the microprocessor, which compares the signal levels against predetermined parameters. One study assessing this particular device indicated that its sensitivity and specificity were superior to DIAGNOdent. The sensitivity and specificity of interproximal detection using the Midwest Caries I.D. compared to X-rays were acceptable [70].

Although it has exhibited a notable degree of sensitivity, conversely, it exhibited a lower level of specificity, thereby presenting a heightened susceptibility to producing false-positive outcomes [71]. It has demonstrated utility in identifying demineralization; however, a significant drawback of this device is its inability to evaluate the extent of demineralization accurately [72].

Alternating Current Impedance Spectroscopy

The CarieScan PRO is a device developed in 2007 that employs alternating current impedance spectroscopy technology. This phenomenon is based on the premise that dental hard tissue demonstrates elevated electrical resistance or impedance, while the resistance decreases as the tissue becomes more demineralized. The device aims to identify and monitor initial coronal dental caries to facilitate early intervention for preventive treatment [61]. However, it showed moderate sensitivity and specificity levels [73].

Cone Beam Computed Tomography (CBCT)

The advent of CBCT in 2001 has brought about a significant breakthrough in dentistry, enabling the acquisition of highly detailed three-dimensional images. The previous study's findings yielded a comparable outcome, indicating that CBCT demonstrated higher sensitivity and overall accuracy in detecting proximal cavitated carious lesions compared to bitewings. However, the specificity between the two methods did not differ significantly [74].

Ultrasonic Method

Ultrasound was first used in 1956 for medical purposes. Ultrasound devices exhibit a greater degree of sensitivity in detecting proximal caries when compared to bitewing radiographs. Nevertheless, a limitation of this methodology is its ability to identify dental caries solely after the tooth reaches a specific level of damage and after alterations in the structure of enamel and dentin have already taken place. In an experimental setting, the ultrasound diagnostic device exhibited superior sensitivity and specificity in detecting approximal carious lesions compared to bitewing radiographs [75].

Developing methods of caries diagnosis and detection

Photothermal Radiometry and Modulated Luminescence (Canary System)

The Canary System introduced in 2013 represents a recent and pioneering advancement in caries detection. The Canary System integrates the technologies of photothermal radiometry (PTR) and luminescence (LUM). In this system, laser light pulses are directed onto the tooth, converting the light into heat and energy. In instances where a lesion is forming, there exists a concomitant alteration in the signal, as the heat (PTR) is localized within the demineralized region, resulting in a reduction in luminosity (LUM). As the process of remineralization takes place, the thermal and LUM properties of the tooth will gradually return to those observed in a healthy tooth structure. The system quantifies the intensity, magnitude, and temporal discrepancy or phase of the transformed thermal energy and emitted light. The signals are processed by an algorithm to generate the Canary Number, which falls from 0 to 100. A Canary Number below 20 is indicative of a healthy tooth structure. The utilization of PTR-LUM technology in the Canary System has demonstrated its capability to identify initial lesions measuring as small as 50 microns and located up to a depth of 5 mm beneath the tooth's surface. The utilization of this technique enables the identification of occlusal pit and fissure caries, smooth surface caries, acid erosion lesions, root caries, and interproximal carious lesions, as well as the assessment of demineralization and remineralization in early carious lesions [76].

Compared to traditional techniques of visual inspection and bitewing radiography in identifying proximal caries, the Canary System exhibited superior sensitivity, with a specificity that was marginally lower than bitewings [77]. The Canary System demonstrated the highest positive and negative predictive values compared to histological examination, which served as the reference standard for determining the presence or absence of carious lesions. A separate investigation utilized the Canary System to identify proximal caries on primary molars. Patients exhibited a favorable tolerance toward the system. Canary System demonstrated a high sensitivity but a comparatively low specificity compared to bitewing radiographs [78].

It has been reported that PTR/LUM values were positively correlated with non-cavitated lesion depth and were unaffected by tissue thickness beneath the lesion despite increasing the scanning angle within 20 degrees [79].

Terahertz Imaging

The first terahertz imaging system was developed in 1995. Terahertz waves, which are electromagnetic waves with frequencies ranging from 0.1 to 10 THz, possess several advantageous properties. These include their non-destructive nature, absence of ionizing radiation hazards, and ability to identify the unique fingerprint spectrum of molecular characteristics. Consequently, terahertz waves exhibit promising potential for extensive biomedicine use [80]. The structural changes and mineral contents of teeth observed using the terahertz imaging system strongly correlate with the findings of X-ray images. Specifically, imaging within the frequency range of 0.4 THz enables the observation of distinct differences between healthy and carious tooth tissue. Based on the findings, it can be inferred that the THz imaging system is an effective technique for evaluating dental caries [48].

Multiphoton Imaging Microscope

Multiphoton imaging microscope was first introduced in 1990. The utilization of this technique has been observed in various studies within the field of dentistry, particularly in the investigation of caries diagnosis and the analysis of structural alterations in tooth enamel [81]. Enamel and dentin can be differentiated through fluorescence lifetime measurement techniques. Multiphoton (two-photon) stimulation has demonstrated enhanced efficacy in imaging due to its ability to achieve deeper penetration, thus offering distinct advantages. The fluorescence lifetime measurement can differentiate between various types of tooth tissue, explicitly distinguishing between carious tooth tissue and healthy tooth tissue [82].

Fluorescence Spectroscopy

Numerous diagnostic techniques have been devised utilizing tooth tissue and its interaction with light. The emergence of novel methodologies, such as fluorescence spectroscopy, has presented an opportunity to detect superficial abnormalities, such as white lesions. Using fluorescence spectroscopy with a 405 nm diode laser, it was discovered that the fluorescence intensity obtained from the outer surfaces of the samples and those emanating from the center of the lesion differ significantly for natural carious lesions and healthy tissue [83].

SOPROLIFE

SOPROLIFE (light-emitting diode fluorescence device) is a caries detection device and a high-magnification intraoral camera in one. SOPROLIFE is software independent. Therefore, it is compatible with most imaging and practice management software. During excavation, the margins of infected and affected dentin are easily distinguishable, allowing the infected dentin (which appears brilliant red) to be removed and the affected dentin (which appears orange) to be retained so that the remaining tooth can recover [36]. One of the notable benefits of utilizing SOPROLIFE is its ability to visually present caries lesions on a computer monitor. This feature facilitates effective follow-up procedures and enhances patient motivation [84].

Soprocare

The Soprocare camera has three distinct clinical applications: daylight, caries, and periodontal. The caries mode primarily targets the occurrence of enamel and dentin caries, whereas the periodontal mode is designed to identify and assess periodontal inflammation [73]. Using the Soprocare camera for examination demonstrates a relatively lower sensitivity but a higher degree of specificity in detecting ICDAS code 1 and 2 lesions [85]. Teledentistry consultations have the potential to demonstrate satisfactory diagnostic performance in detecting dental caries. The Soprocare camera facilitates the timely detection of carious lesions with optimal efficacy [86].

Sapro Imaging Software

The Sapro imaging software is a computer program designed to store and archive captured images, allowing users to compare them later conveniently. The software can save the pictures produced by positioning the camera on the tooth after selecting the desired magnification and mode (daylight or fluorescent) [73].

Raman Spectroscopy

Raman spectroscopy (RS) is a highly effective label-free technique that can quantitatively assess the biomolecular composition of tissues. RS is a technique that utilizes light to analyze the molecular bonds of materials through the scattering of photons. RS can provide information regarding the lesion's molecular structure. When a material is exposed to monochromatic light of varying wavelengths, specific photons within the light experience a frequency shift and scatter. This phenomenon is contingent upon the molecular bonds within the material in either the vibrational or rotational phase [87].

RS techniques have facilitated the detection of caries in enamel before the manifestation of visible changes. RS possesses the requisite sensitivity and selectivity to discern early-stage carious lesions effectively. However, the analysis may be complicated by a surface layer exhibiting a comparatively elevated mineral content [88,89]. Due to its high sensitivity and specificity, polarized RS is a potential tool for detecting early dental caries and monitoring the progress of the lesion after fluoride treatment [90].

Confocal Laser Scanning Microscope

Laser scanning is a technique that utilizes laser beams to capture and measure the physical characteristics of objects or environments in a three-dimensional manner. The confocal microscope, which falls under the category of laser scanning microscopes, offers the capability to capture high-resolution images of live organisms. It can also generate three-dimensional representations of tissues up to 1 mm thick by producing vertical sections within the specific tissue of interest [91]. The sample is scanned in lines using a focused laser source illumination. Excluding reflections originating from outside the designated area solely monitors the concentrated region. The measurement of porosities within the demineralized enamel layer is conducted through infiltrating fluorescent dyes [92]. The confocal laser scanning microscope is an invaluable instrument in dental research, specifically for examining dense samples of carious teeth and various dental research involving live tissue imaging [93].

Deep Learning-Based Convolutional Neural Network Algorithm

Recently, a notable area of focus within the field of artificial intelligence and deep learning has been convolutional neural networks (CNNs). These networks have exhibited remarkable capabilities in computer vision, specifically in tasks such as object, facial, and activity recognition, as well as tracking and three-dimensional mapping and localization [94]. Deep CNN algorithms efficiently perform edge detection by utilizing multiple convolutional and hidden layers to create hierarchical feature representations. Consequently, a deep CNN-based dental caries detector can effectively learn dental carious lesions' location and morphological alterations. This enables the detector to detect such lesions conveniently and reliably [95]. Ongoing advancements in deep learning algorithms, such as ResNet and CapsNet, have led to the development of models with increased depth or width or modified layering techniques. Consequently, these advancements have consistently and significantly enhanced the accuracy of object detection and segmentation [96,97]. The CapsNet model, a recently developed framework, has demonstrated significant utility in analyzing visual attributes such as posture (including location, size, and direction), modification, speed, reflection coefficient, hue, texture, and encoding [97].

## Conclusions

The transition toward minimally invasive dentistry has allowed dentists to identify and address caries in its early stages. Several technologies have been developed to detect lesions at different stages of the caries process. These technologies include optical, radiographic, electrical, ultrasound, and artificial intelligence-based deep learning technologies. The visual assessment method continues to be the widely accepted and fundamental approach to assessment, which is quick and cost-effective with lesion detection accuracy. Emerging caries detection devices have the potential to assist dental professionals in identifying, quantifying, and monitoring lesions over time. Fluorescence and transillumination methods utilizing optical technology have been identified as highly effective techniques for detecting early-stage carious lesions.
